# SIRT1/PGC-1α Signaling Promotes Mitochondrial Functional Recovery and Reduces Apoptosis after Intracerebral Hemorrhage in Rats

**DOI:** 10.3389/fnmol.2017.00443

**Published:** 2018-01-09

**Authors:** Yang Zhou, Shaohua Wang, Yixin Li, Shanshan Yu, Yong Zhao

**Affiliations:** ^1^Department of Pathology, Chongqing Medical University, Chongqing, China; ^2^Institute of Neuroscience, Chongqing Medical University, Chongqing, China

**Keywords:** SIRT1, PGC-1α, intracerebral hemorrhage, mitochondrial biogenesis, apoptosis

## Abstract

Silent information regulator 1 (SIRT1) exerts neuroprotection in many neurodegenerative diseases. However, it is not clear if SIRT1 has protective effects after intracerebral hemorrhage (ICH)-induced brain injury in rats. Thus, our goal was to examine the influence of SIRT1 on ICH injuries and any underlying mechanisms of this influence. Brain injury was induced by autologous arterial blood (60 μL) injection into rat brains, and data show that activation of SIRT1 with SRT1720 (5 mg/kg) restored nuclear SIRT1, deacetylation of PGC-1α, and mitochondrial biogenesis and decreased mortality, behavioral deficits, and brain water content without significant changes in phosphorylated AMP-activated protein kinase (pAMPK) induced by ICH. Activation of SIRT1 with SRT1720 also restored mitochondrial electron transport chain proteins and decreased apoptotic proteins in ICH; however, these changes were reversed after ICH. In contrast, treatment with PGC-1α siRNA yielded opposite effects. To explore the protective effects of SIRT1 after ICH, siRNAs were used to knockdown SIRT1. Treatment with SIRT1 siRNA increased mortality, behavioral deficits, brain water content, mitochondrial dysfunction, and neurocyte apoptosis after ICH. Thus, activation of SIRT1 promotes recovery of mitochondrial protein and function by increasing mitochondrial biogenesis and reduces apoptosis after ICH via the PGC-1α mitochondrial pathway. These data may suggest a new therapeutic approach for ICH injuries.

## Introduction

Sudden intracerebral hemorrhage (ICH) is a catastrophic stroke subtype with high morbidity and mortality (Keep et al., 2012) and no effective pharmacological treatment due to deficits in understanding the underlying mechanisms of post-ICH brain injury. Many types of pathophysiology occur after ICH, but neuronal apoptosis is considered significant (Qureshi et al., 2001). In mammals, apoptosis occurs via two main pathways: the death receptor pathway (extrinsic apoptotic pathway) and the mitochondrial pathway (intrinsic apoptotic pathway) (Green, 2000). Mitochondria are the main source of ATP in eukaryotic cells, and mitochondria are chief sites of apoptosis essential to initiating apoptotic cell death (Giorgi et al., 2012). Recent studies suggest increased mitochondrial dysfunction occurs in the perihematomal region after ICH (Kim-Han et al., 2006), and similar results were observed in brain tissue obtained from acute traumatic brain injury patients undergoing surgical intervention (Verweij et al., 2000), indicating that mitochondrial dysfunction is associated with brain injury after ICH. However, how mitochondrial dysfunction and mitochondrial-dependent apoptosis function in ICH pathogenesis is not clear.

Silent information regulator 1 (SIRT1), an NAD+-dependent protein deacetylase, is involved in different biological processes, such as inflammation, cell death, and metabolism, by regulating main targets through deacetylation (Imai et al., 2000). Previous studies have shown that activation of SIRT1 plays an important neuroprotective role in neurodegenerative diseases (Kim et al., 2007; Mudo et al., 2012). SIRT1 deacetylates histone and numerous non-histone proteins during transcription, including peroxisome proliferator initiated receptor gamma and coactivator 1 alpha (PGC-1α) (Lagouge et al., 2006). PGC-1α is a nuclear transcriptional co-activator of nuclear receptors and other transcription factors and is strongly expressed in the heart, kidney, and brain and in skeletal muscle and brown adipose tissue (Shi and Gibson, 2007). Moreover, PGC-1α is a central regulator of mitochondrial biogenesis that can promote biosynthesis of mitochondria. Activation of PGC-1α could improve the mitochondrial biogenesis in AD (Makela et al., 2016). Of note, SIRT1 interacts with and deacetylates PGC-1α to enhance its activity, promote mitochondrial biogenesis, and maintain mitochondrial function (Lagouge et al., 2006). Previous studies have shown that activation of SIRT1/PGC-1α pathway play a protective role in neuronal injuries induced by the neurotoxin MPTP (Mudo et al., 2012). Also, SIRT1 suppression decrease PGC-1α in ischemic heart disease (Hu et al., 2016). Researchers suggest that mitochondrial apoptosis is reduced after PGC-1α overexpression in human sarcoma cell lines (Onishi et al., 2014), indicating that SIRT1 may be protective during mitochondrial-dependent apoptosis. Currently, alterations in SIRT1 and localization have been linked to cardiac ischemia/reperfusion damage, hepatic ischemia/reperfusion damage, and hepatic lipid homeostasis (Wang et al., 2010; Li et al., 2011; Rickenbacher et al., 2014; Hu et al., 2016). In addition, SIRT1 activity changes with its subcellular location. The literature suggests that nuclear SIRT1 may increase its activity by deacetylating PGC-1α.

Considering the effects of SIRT1 on mitochondrial dysfunction and apoptosis, we studied mechanisms underlying the function of SIRT1 in ICH and noted that nuclear SIRT1 decreased, and total SIRT1 increased, in rats subjected to ICH. SIRT1 activation halts ICH-induced brain injury by improving mitochondrial biogenesis and decreasing mitochondrial-dependent apoptosis via deacetylation of PGC-1α *in vitro*. In contrast, knockdown of SIRT1 with siRNA worsened injuries after ICH in rats.

## Materials and Methods

### Animals and Grouping

Male Sprague-Dawley rats (260–310 g) were purchased from the Experimental Animal Center of Chongqing Medical University (Chongqing, China) and housed under a 12-h light/dark cycle with unlimited food and water. All rat care and use procedures complied with established regulations at Chongqing Medical University and performed with the consent of the Institutional Animal Care and Use Committee (IACUC) and in conformance with the NIH Guidelines for the Care and Use of Laboratory Animals.

A total of 313 rats were randomized to 8 groups: (1) sham (*n* = 20), (2) ICH (*n* = 38; 6 died), (3) ICH + vehicle (ICH + V) (n = 42; 6 died), (4) ICH + SRT1720 (*n* = 34; 4 died), (5) ICH + negative control siRNA (ICH + NC) (*n* = 43; 3 died), (6) ICH + SRT1720 + PGC-1α siRNA (*n* = 36; 5 died), (7) ICH + PGC-1α siRNA (*n* = 30; 4 died), and (8) ICH + SIRT1 siRNA (*n* = 36; 6 died).

### ICH Model and SRT1720 Dosing

Rats were initially anesthetized with chloral hydrate (10% solution, ip), and then procedures were conducted as described in the literature with slight modifications (Xue and Del Bigio, 2000). Briefly, autologous blood (60 μL) was drawn into an aseptic syringe from the femoral artery. The aseptic syringe was then inserted stereotaxically into the right lower ganglia (coordinates of 0.2 mm anterior, 5.5 mm ventral, and 3.5 mm lateral to the bregma) (Yang et al., 2008; Li et al., 2013). Autologous blood was administered at 10 mL/min. After 10 min, the needle was removed; the incision was sutured; and the rat was allowed to recuperate. Sham-treated rats were administered the same volume of saline. Experimental animals were killed 48 h after ICH.

SRT1720 was produced as described previously (Milne et al., 2007; Funk et al., 2010). The Sirt1 activator Sirt1 agonist SRT1720 (S1129, Selleck) was dissolved in DMSO and diluted to a final concentration with normal saline (final DMSO < 1%). We intracranially injected SRT1720 into rats after the onset of ICH (5 mg/kg/d) before sacrifice 48 h later. An identical volume of DMSO was injected intracranially as a control.

### Tissue Preparation and Histological Evaluation

After neurological evaluation, animals were killed using an overdose of 3.5% chloral hydrate at 48 h after ICH, and brains were perfused transcardially using 0.9% sodium chloride followed by 4% paraformaldehyde. After decapitation, the brains were dissected and embedded in paraffin and samples from all groups were assessed. Specifically, 5-μm coronal sections 1.2 mm in the front and 3.6 mm in the back of the bregma were stained with 0.1% cresyl violet hematoxylin and eosin (H&E) based on standard procedures and prepared for microscopic examination (Xu et al., 2006). H&E data were assessed by pathologists who assessed neuronal morphological features. Briefly, they visualized a large round nucleus in the central portion of neurons with less heterochromatin, lighter staining, and large nucleoli. Nissl bodies observed were large or granular basophilic substances in H&E stained sections.

### Brain Water Content Assay

To determine brain water content, cerebral edema was assessed using wet weight (WW) to dry weight (DW) ratios as previously described (Chu et al., 2004). In brief, after ICH, rats were sacrificed at designated time points, and contralateral and ipsilateral hemispheres and cerebellums were quickly removed to obtain the WW. Cerebellums served as internal controls. Tissue was dried in a 100°C oven for 24 h and then weighed again to acquire DW. Tissue water content was calculated as: (WW - DW)/WW × 100%.

### Garcia Neurological Score Assay

Intracerebral hemorrhage rats were scored lowest to highest (0–18) in the Garcia exam, which included unplanned pursuits, axial sensation, vibrissae proprioception, appendage outstretching, lateral turning, and forelimb walking. For each part, the rat scored 0–3 (0 = the worst; 3 = the best). Greater scores indicated better neurological function.

### TUNEL Staining

TUNEL staining was performed according to the manufacturer’s instructions (11684817910, Roche Molecular Biochemicals, Inc., Mannheim, Germany). Briefly, sections were treated with Proteinase K (20 μg/mL for 15 min) and 0.3% H_2_O_2_ for 30 min. Then, sections were incubated with terminal deoxyribonucleotidyl transferase (TdT) enzyme at 37°C for 1 h and incubated with peroxidase-conjugated antibody for 30 min at 37°C. Sections were allowed to react with 3,3′-diaminobenzidine tetrahydrochloride solution for 10 min at room temperature. Under a fluorescent microscope (Olympus BX51, Japan), TUNEL-positive cells identified by green fluorescence of FITC were counted in 10 microscopic fields of each brain section at 400x magnification. An apoptosis index was counted as apoptotic nuclei within 100 nuclei. The mean apoptosis index (AI) = TUNEL-positive cells/total cell number × 100%.

### Treatment with SIRT1 or PGC-1α siRNA

All siRNAs were designed and synthesized by GenePharma Corporation, Shanghai, China. RNAi experimental processes included target gene identification, siRNA design, siRNA generation, siRNA transfection, measurement of knockdown, and functional assessment. Specific siRNA that targeted a gene and negative control siRNA that was non-specific were used. Interference efficiency of a specific siRNA was confirmed with Western blot and real-time PCR. For *in vivo* siRNA administration, specific siRNA targeting against SIRT1 (with the targeting sequence: sense primer 5′-GCCACCAACACCUCUUCAUTT-3′ and antisense primer 5′-AUGAAGAGGUGUUGGUGGCTT-3′) or PGC-1α (with the targeting sequence: sense primer 5′-GCUC UUGAGAAUGGAUAUATT-3′ and antisense primer 5′-UAUA UCCAUUCUCAAGAGCTT-3′) (GenePharma Corporation, location?) was administered 24 h pre-ICH via intracerebroventricular injection as described previously (Ma et al., 2014). Negative control siRNAs had no sequence homology to recognized rat genes. A cranial burr hole (1 mm) was made, and a 30-gauge needle was placed stereotaxically into the right lateral ventricle. To increase gene silencing function, siRNAs were dissolved in DEPC water. SIRT1 siRNA, PGC-1α siRNA, or control siRNA was administered intracerebroventricularly for 5 min. The needle was kept in place for an additional 10 min post-injection and then slowly removed. After 24 h, all ICH models were established as described above.

### Western Blot

To acquire specimens for Western blot, brains were dissected at 48 h post-ICH and then perfused with cold saline. Brain tissue surrounding the hematoma (except for the hematoma area and 3 mm beyond) and a uniform portion of the contralateral tissue were anatomized and immediately frozen at -80°C until use. Cell cytosol was isolated with the BioVision fractionation kit (K256-100, Mountain View, CA, United States) according to the manufacturer’s protocol. Total protein content was quantified using a BCA assay (Beyotime, Jiangsu, China). A total of 50 μg of protein was separated using SDS-PAGE, and immunoblots were processed as described previously (Rasbach and Schnellmann, 2007). GAPDH was used as a loading control for total lysate immunoblots. Antibodies used were as follows: SIRT1 (1:500, Cell Signaling Technologies, Danvers, MA), PGC-1α (1:1,000, Abcam, Cambridge, MA, United States), AMP-activated protein kinase (AMPK) (1:1,000, Proteintech), phosphorylated AMP-activated protein kinase (pAMPK) (1:500, Bioworld), acetylated-lysine (1:500, Bioworld), lamin B1 (Cell Signaling Technologies), GAPDH (1:2000, Bioworld), ATP synthase β (ATPβ) (1:400, Bioworld), cytochrome c oxidase subunit I (1:400, Bioworld), NADH dehydrogenase (ubiquinone) 1 beta subcomplex subunit 8 (NDUFB8) (1:400, Bioworld), cytochrome c (1:1,000, Abcam), AIF (1:1,000, Santa Cruz, Dallas, TX), cleaved caspase-3 (cleaved c3) (1:1,000, Cell Signaling Technologies, Danvers, MA), and caspase-3 (c3) (1:500, Santa Cruz). Nuclear lysates were prepared as previously described (Funk et al., 2010). Briefly, brain tissue was homogenized in sucrose buffer (50 mM Tris-HCl, 1 mM β-mercaptoethanol, 1 mM EDTA, and 320 mM sucrose) using a pestle and sonication (10 s). Lysates were then centrifuged at 900 × *g* for 10 min, and nuclear pellets were resuspended in RIPA solution. Lamin B1 was used as a loading control for nuclear lysate immunoblots.

### Quantitative Real-Time PCR (QPCR)

Forty-eight hours after ICH, brain tissue around the hematoma (excluding the hematoma area and extending 3 mm beyond the hematoma) and a uniform portion of the contralateral tissue were obtained. Total RNA was isolated with RNAiso Plus reagent (TaKaRa Biotechnology Co., Ltd., China) according to kit instructions. mRNA extracted was measured using real-time PCR with SYBR PremixEx Taq II (Tli RNaseH Plus) (TaKaRa Biotechnology Co.). PGC-1α gene expression for each sample was normalized to expression of GAPDH. QPCR reaction conditions were as follows: 1 cycle of 94°C for 5 min, 40 cycles of 94°C for 30 s, 58°C for 30 s, and 72°C for 30 s. The primer sequences were as follows: SIRT1 (forward primer, 5′-GTCTGTGCCTTCCAGTTGCT-3′, reverse primer, 5′-CTGCTTGCTGTCCATACCTG-3′); PGC-1α (forward primer, 5′-ACATCgCAATTCTCCCTT-3′, reverse primer, 5′-CTCTTgAgCCTTTCgTgCTC-3′), and GAPDH (forward primer, 5′-ACAGCAACAGGGTGGTGGAC-3′, reverse primer, 5′-TTTGAGGGTGCAGCGAACTT-3′). Primers were synthesized by Sangon Biotech, Shanghai, China. Each reaction was performed a minimum of three times.

### Mitochondrial DNA (mtDNA)

Total DNA from specimens (*n* = 6/group) was isolated using the TIANamp Genomic DNA Kit (Tiangen Biotech) according to the manufacturer’s instructions. We quantified mtDNA content using real-time PCR with 10 ng of total DNA with primers that amplify the D-loop area. The D-loop primer sequences were as follows: forward primer, 5′-AATCTACCATCCTCCGTG-3′ and reverse primer, 5′-GACTAATGATTCTTCACCGT-3′ (Sangon Biotech).

### HPLC

Next, 48 h after ICH, brain tissue specimens from the ipsilateral side of the cerebral cortex were sonicated in 15 volumes (wt/vol) of saline consisting of 60% acetonitrile for 5–10 s. After centrifugation at 14,000 × *g* for 15 min at 4°C, Supernatant was diluted with 30 mM phosphate solution and evaluated with an Agilent 1200 HPLC system (Agilent) using an LC-18 column (15 mm × 4.6 mm, 5 μm, Supelco Discovery, Bellefonte, PA, United States) and a moving phase flow rate of 1.0 mL/min. The detection wavelength was set at 254 nm. The Agilent1200 HPLC system included a G1311A quaternary pump, a G1329A self-activating sampler, a G13f14B differing-wavelength detector, and an Agilent 1200 station. The elution peak of the specimen was validated by comparing it with ATP standards (Sigma–Aldrich).

### Projection Electron Microscopy

At 48 h post-ICH, the rats were deeply anesthetized and intracardially perfused with 0.9% saline followed by a solution of 4% paraformaldehyde mixed with 2.5% glutaraldehyde in PBS. Ipsilateral striatal tissue samples were cut into 1-mm^3^ pieces. We arbitrarily chose 15 samples from each rat imaged at 50,000x magnification to assess mitochondrial ultrastructure.

### Immunoprecipitation and PGC-1α Acetylation

Immunoprecipitation was performed as described previously based on the company’s directions (Roche Applied Science, Indianapolis, IN, United States). Briefly, 500 μg total protein from total tissue lysates or nuclear lysates was incubated with protein A-agarose beads (BD0045, Bioworld Technology, Inc.) for 4 h to vacate specimens. After centrifugation, the supernatant was removed and incubated with anti-PGC-1α antibody overnight at 4°C with slight agitation. Lysates were then incubated with protein A-agarose beads for 3 h at 4°C. Post-cleansing, the beads were boiled to free the product, which was separated by SDS-PAGE and immunoblotted for acetylated lysine. Band visualization as performed as described. Membranes were stripped using 0.1M glycine (pH 2) for 20 min and reprobed with anti-PGC-1a antibody.

### Data Analysis and Statistics

All data are shown as means ± SEM, and statistical analysis was conducted with GraphPad Prism 6.0 and SPSS 18.0. Multiple comparisons among vehicle and treatment groups were performed using one-way ANOVA followed by Bonferroni correction. Differences were considered statistically significant when *p* < 0.05.

## Results

### SIRT1 Expression and Localization after ICH and SRT1720 Treatment

**FIGURE 1:**
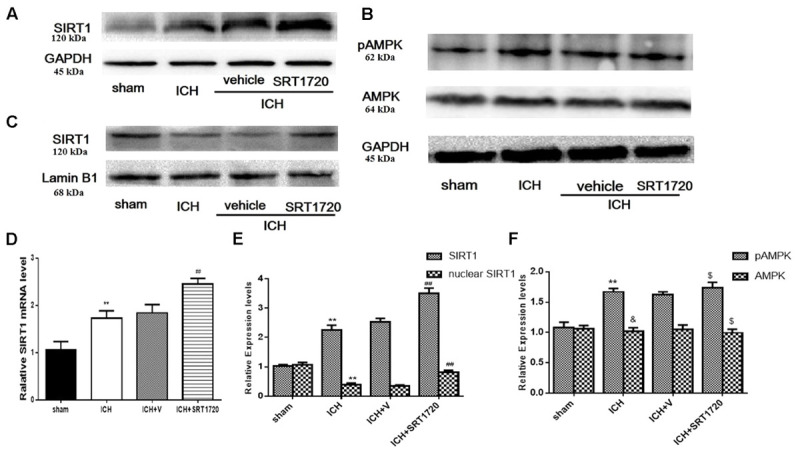
SIRT1 expression and localization after ICH and SRT1720 treatment. Representative Western blots of **(A)** total SIRT1, **(B)** pAMPK and AMPK, **(C)** nuclear SIRT1. **(D)** QPCR results of SIRT1. Relative protein band density values of total SIRT1, pAMPK, and AMPK were computed as the ratio of protein of interest to that of GAPDH. Relative protein band density value of nuclear SIRT1 was computed as the ratio of protein of interest to that of lamin B1. Quantification of **(A–C)** is shown in **(E,F)**, respectively. Error bars show means ± SEM. (^∗∗^*P* < 0.01 vs. sham; ^##^*P* < 0.01 vs. ICH; ^&^*P* > 0.05 vs. sham; ^$^*P* > 0.05 vs. ICH). *n* = 4 in sham group, and *n* = 6 in other groups.

Since SIRT1 activators may act on AMPK as well as SIRT1 (Baur et al., 2006; Dasgupta and Milbrandt, 2007), we evaluated levels of pAMPK, AMPK, and SIRT1. pAMPK increased in the ICH and ICH + vehicle groups; however, there was no effect of SRT1720 treatment on pAMPK levels (**Figures 1B,F**). Overall AMPK did not change with treatment or post-ICH (**Figures 1B,F**). SRT1720 functions as a specific activator of SIRT1. To examine the efficiency of SRT1720 on SIRT1, western blotting and QPCR were performed to determine SIRT1 protein and mRNA levels 48 h after ICH (**Figures 1A,C–E**). Compared with shams, total SIRT1 increased post-ICH, and SRT1720 treatment post-ICH caused even further increases in SIRT1 (**Figures 1A,D,E**). Immunoblot analysis of nuclear lysates revealed that compared with shams, there was an apparent reduction of nuclear SIRT1 in the ICH group, which was restored to normal levels after treatment with SRT1720 (**Figures 1C,E**).

### Brain Water Content and Neurological Outcomes 48 h after ICH Injury after SIRT1 Activation

**FIGURE 2:**
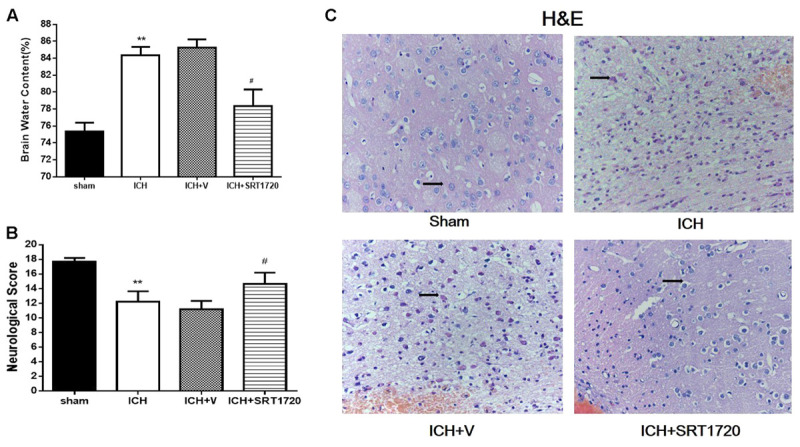
Brain water content, neurological outcomes, and histological assessment 48 h after ICH injury after SIRT1 activation in rats. **(A)** Brain water content measured 48 h after ICH in the ipsilateral hemisphere in sham, ICH, ICH + vehicle, and ICH + SRT1720 groups. **(B)** Neurological scores of each group. **(C)** HE staining (400x). Arrows indicate neurons around hematomas of ICH rats. Error bars represent means ± SEM. (^∗∗^*P* < 0.01 vs. sham; ^#^*P* < 0.05 vs. ICH). For **(A,C)**, *n* = 4 sham group, and *n* = 6 in other groups. For **(B)**, *n* = 20 in sham group, and *n* = 30 in other groups.

Brain water content 48 h after ICH (**Figure 2A**) and was substantially higher in the ipsilateral hemicerebrum in the ICH group compared to shams but activation of SIRT1 with SRT1720 reduced brain water content compared with the ipsilateral hemicerebrum in the ICH group. There was a significant decrease in neurological scores in the modified Garcia test in the ICH group compared with shams (**Figure 2B**) and treatment with SRT1720 48 h after ICH significantly improved neurobehavioral function in the modified Garcia test compared with the ICH group.

### Histology 48 h after ICH Injury after SIRT1 Activation

H&E staining to assess morphological changes in the hemorrhagic lesions 48 h after ICH (**Figure 2C**) showed that compared to shams, neurocytes in the ICH and ICH + vehicle groups were disordered and had shrinking cell bodies, nuclear pyrosis and fragmentation, and there were more red neurons. In addition, ICH increased diffuse vacuolization and edema in interstitial areas. Nevertheless, activation of SIRT1 alleviated diffuse vacuolization and edema in interstitial areas, and SRT1720 substantially decreased degraded neurons and increased total neurons.

### SIRT1 Activation Induced Deacetylation of PGC-1α after ICH

**FIGURE 3:**
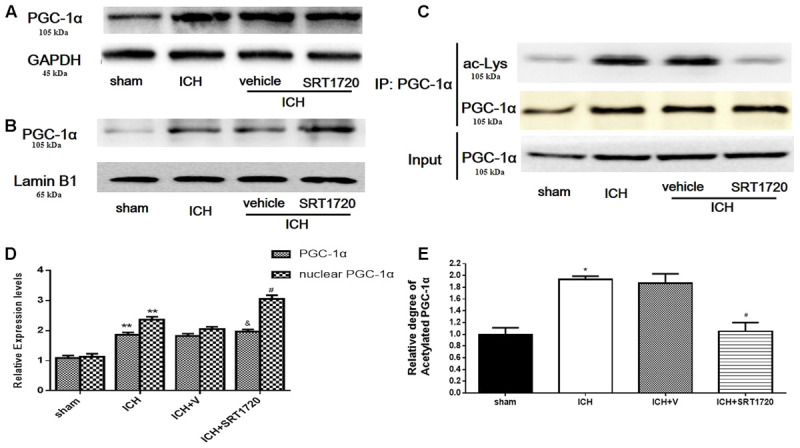
SIRT1 activation induced deacetylation of PGC-1α after ICH. **(A)** Total PGC-1α protein in brain tissue 48 h after ICH assessed with immunoblot. Relative protein band density value of total PGC-1α was calculated as the ratio of protein of interest to that of GAPDH. **(B)** Nuclear PGC-1α 48 h after ICH assessed with immunoblot. Relative protein band density value of nuclear PGC-1α was calculated as the ratio of protein of interest to that of lamin B1. **(C)** Acetylation of PGC-1α 48 h after ICH as measured by immunoprecipitation. Quantification of **(A–C)** is shown in **(D,E)**, respectively. Error bars represent mean ± SEM. (^∗∗^*P* < 0.01 vs. sham; ^#^*P* < 0.05, ^##^*P* < 0.01 vs. ICH; ^&^*P* > 0.05 vs. ICH). *n* = 4 in sham group, and n = 6 in other groups.

Compared with shams, PGC-1α protein was upregulated at 48 h in the ICH and ICH + vehicle groups (**Figures 3A,D**); however, we did not detect significant differences between the ICH and ICH + SRT1720 groups. Because changes in nuclear localization of PGC-1α can affect its function and activation of SRIT1 with SRT1720 can increase expression of nuclear PGC-1α (Funk et al., 2010), we examined protein levels of PGC-1α in nuclear lysates. Similar to results of Funk and colleagues, PGC-1α levels increased in nuclear lysates after treatment with SRT1720 (**Figures 3B,D**). Since SIRT1 is a protein deacetylase that targets PGC-1α (Nemoto et al., 2005), PGC-1α acetylation (Ac-PGC-1α) was evaluated in lysates after ICH and SRT1720 treatment (**Figures 3C,E**). Brain lysates were subjected to immunoprecipitation with a PGC-1α antibody followed by immunoblot evaluation with an antibody to acetylated-lysine. We noted an increase in Ac-PGC-1α in the ICH group and normal levels of Ac-PGC-1α in the ICH + SRT1720 group. These results suggest that most PGC-1α in the ICH + SRT1720 group post-injury was deacetylated compared with ICH group.

### ATP and Mitochondrial DNA Content after ICH Injury after SIRT1 Activation

**FIGURE 4:**
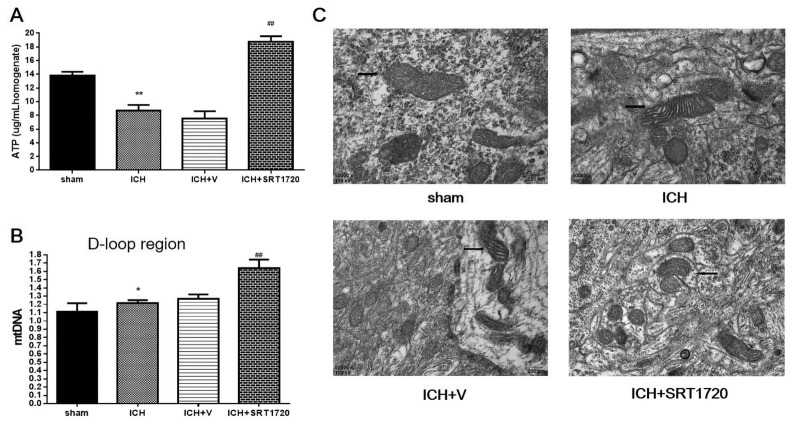
ATP concentration, mitochondrial DNA content, and mitochondrial structural changes after ICH injury after SIRT1 activation in rats. **(A)** Changes in ATP concentration in the brain tissue 48 h after ICH assessed with HPLC with a variable wavelength detector (HPLC-VWD). **(B)** Real-time PCR of the D-loop area of mtDNA in brain tissue 48 h after ICH. **(C)** Mitochondrial structural changes assessed by projection electron microscopy (50,000x). Arrows indicate mitochondria around hematomas of ICH rats. Error bars represent means ± SEM. (^∗^*P* < 0.05, ^∗∗^*P* < 0.01 vs. sham; ^##^*P* < 0.01 vs. ICH). *n* = 4 in sham group, and n = 6 in other groups.

Because mitochondria are the primary source of ATP, we measured ATP with HPLC (**Figure 4A**) and ATP was substantially reduced in the ICH group compared with shams. In contrast, ATP was significantly increased in the ICH + SRT1720 group compared with the ICH group.

Mitochondrial biogenesis was assessed by measuring mitochondrial DNA copy number, quantified by QPCR of the mtDNA D-loop region (**Figure 4B**). Compared with shams, mtDNA increased in the ICH group. Also, activation of SIRT1 increased mtDNA compared with the ICH group.

### Mitochondrial Structure after ICH Injury after SIRT1 Activation

Mitochondria respond to different types of injury and show this injury morphologically. Therefore, a projection electron microscope was used to assess mitochondrial ultrastructure (**Figure 4C**). Data show that mitochondrial outer compartment widening was substantially greater in the ICH group than in shams. However, SRT1720 treatment reduced swelling of the mitochondria compared with the ICH group.

### SIRT1 Activation Restored Mitochondrial Electron Transport Chain Protein Expression after ICH via PGC-1α

**FIGURE 5:**
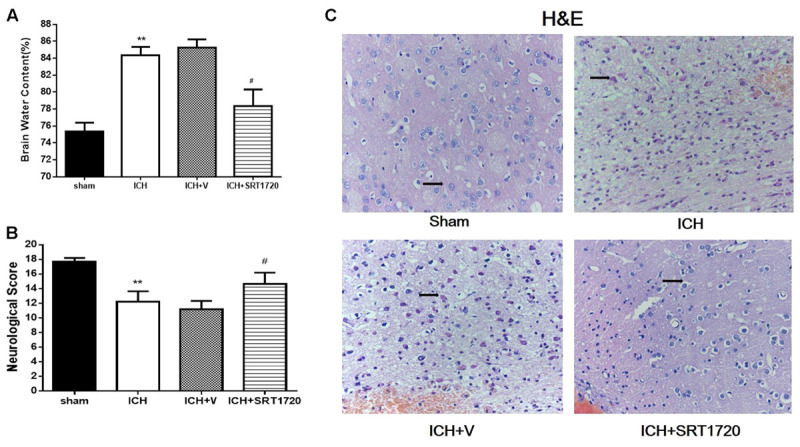
SIRT1 activation restored mitochondrial electron transport chain protein expression after ICH via PGC-1α in rats. Representative Western blots of ATPβ, COX I, NDUFB8 **(A,D)**, and PGC-1α **(B)**, and QPCR results of PGC-1α **(C)**. Relative protein band density values were calculated as the ratio of the protein of interest to that of GAPDH. Quantification of **(A,B,D)** is shown in **(E–G)**, respectively. Error bars represent mean ± SEM. **(C,E,F)** (^∗∗^*P* < 0.01 vs. sham; ^#^*P* < 0.05, ^##^*P* < 0.01 vs. ICH); **(G)** (^∗∗^*P* < 0.01 vs. ICH; ^##^*P* < 0.01 vs. ICH + SRT1720). *n* = 4 in the sham group, and *n* = 6 in the other groups.

Because SIRT1 can regulate PGC-1α, we evaluated expression of mitochondrial electron transport chain proteins after ICH injury (**Figures 5A,F**). Compared with shams, nuclear-encoded proteins ATPβ, NDUFB8, and cytochrome c oxidase subunit I (COX I) were decreased after ICH. Activation of SIRT1 restored expression of mitochondrial electron transport chain protein compared with the ICH group (**Figures 5A,F**). To clarify the protective role of SIRT1 activation in modulating mitochondrial electron transport chain proteins, PGC-1α siRNA was used to decrease expression of PGC-1α. To verify the effect of siRNA on PGC-1α expression, PGC-1α protein and mRNA were measured using Western blot and QPCR, respectively. **Figures 5B,C,E** show that protein and mRNA levels were reduced after treatment with PGC-1α siRNA. As expected, knockdown of PGC-1α eliminated recovery of mitochondrial electron transport chain protein expression after treatment with SRT1720 (**Figures 5D,G**).

### Neuronal Cell Apoptosis of Rats 48 h after ICH after SIRT1 Activation Measured Using TUNEL

**FIGURE 6:**
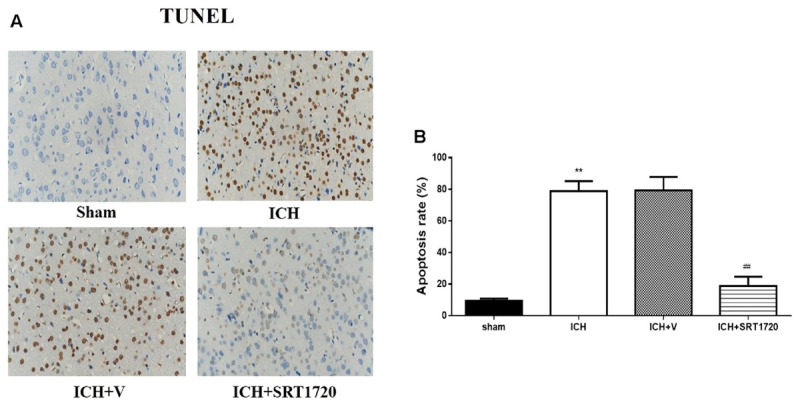
Cell apoptosis of rats at 48 h after ICH following SIRT1 activation detected by TUNEL. **(A)** The TUNEL staining image for rats (×400). **(B)** cell apoptotic rate of rats. Error bars represent mean ± SEM. (^∗∗^*P* < 0.01 vs. sham; ^##^*P* < 0.01 vs. ICH). *n* = 4 in the sham group, and *n* = 6 in the other groups.

TUNEL was used to assess apoptosis of neuronal cells (**Figures 6A,B**). The karyon of normal neuronal cells was uniformly stained blue and advanced apoptotic cell nuclei were brown/dark brown and karyopyknosis was irregular in shape. Cell nuclei were broken and edge set under higher magnification. TUNEL results confirmed more apoptotic neuronal cells in the ICH groups compared to shams. Compared with apoptotic neuronal cells in ICH groups, apoptotic neuronal cells were reduced in the ICH + SRT1720 groups, suggesting that SIRT1 activation reduced apoptosis of neuronal cells.

### Expression Apoptotic Proteins after ICH Injury after SIRT1 Activation

**FIGURE 7:**
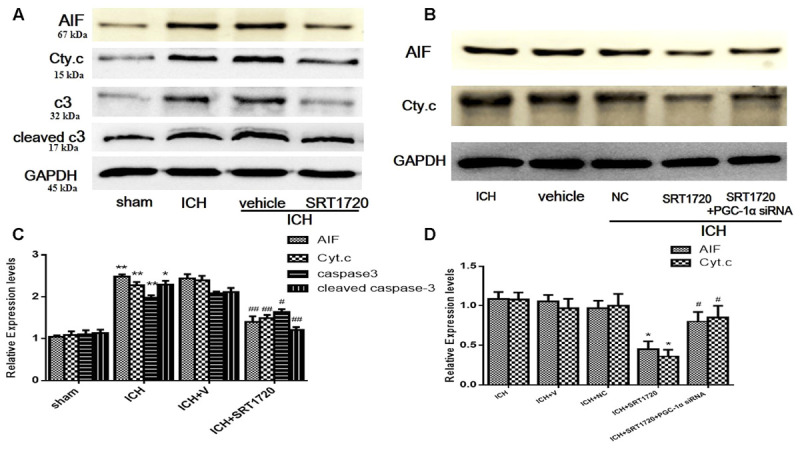
Expression levels of apoptotic proteins after ICH injury following SIRT1 activation in rats. Representative western blots of AIF, cytochrome c, caspase-3 (c3), and cleaved caspase-3 (cleaved c3) **(A,B)**. Relative protein band density values were calculated as the ratio of the protein of interest to that of GAPDH. Quantification of **(A,B)** is shown in **(C,D)**, respectively. Error bars represent mean ± SEM. **(C)** (^∗^*P* < 0.05, ^∗∗^*P* < 0.01 vs. sham; ^#^*P* < 0.05, ^##^*P* < 0.01 vs. ICH); **(D)** (^∗^*P* < 0.05 vs. ICH; ^#^*P* < 0.05 vs. ICH + SRT1720). *n* = 4 in the sham group, and *n* = 6 in the other groups.

To evaluate the role of SIRT1 in regulating apoptosis, apoptotic proteins were quantified with Western blot. Compared with shams, ICH increased cytoplasmic release of cytochrome c and cytoplasmic caspase-3 (c3), cleaved caspase-3 (cleaved c3) and AIF proteins (**Figures 7A,C**). Treatment with SRT1720 reduced these increases in protein expression compared with the ICH group. Thus, activation of SIRT1 has an anti-apoptotic function after ICH. To elucidate the protective role of SIRT1 activation in regulating mitochondria-dependent apoptotic proteins, PGC-1α siRNA was used to decrease PGC-1α. As shown in **Figures 7B,D**, knockdown of PGC-1α increased expression of cytochrome c and AIF after treatment with SRT1720.

### Brain Water Content and Neurological Outcomes 48 h after ICH Injury after SIRT1 Silencing

**FIGURE 8:**
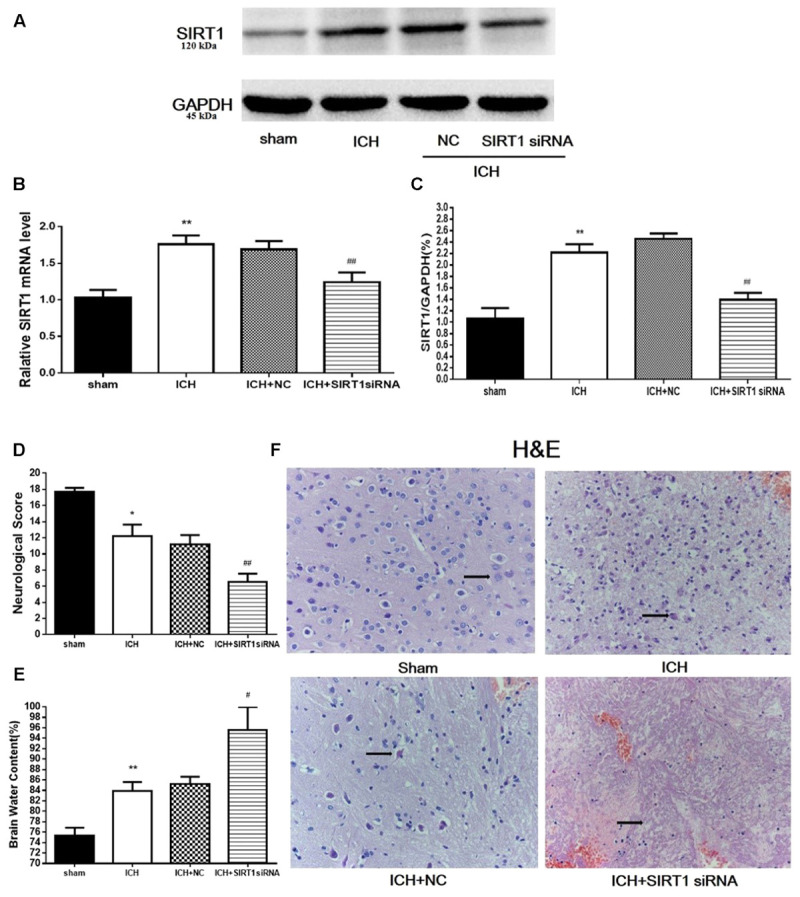
Brain water content, neurological outcomes, and histological assessment at 48 h after ICH injury following SIRT1 silencing in rats. **(A)** Western blots of SIRT1. **(B)** QPCR results of SIRT1. **(C)** Relative protein band density value was calculated as the ratio of the protein of interest to that of GAPDH. Quantification of **(A)** is shown in **(C)**. **(D)** Neurological scores and **(E)** brain water content of each group at 48 h after ICH. **(F)** Representative HE staining of each group (×400). Arrows indicate neurons around the hematoma of rats with ICH. Error bars represent mean ± SEM. (^∗^*P* < 0.05, ^∗∗^*P* < 0.01 vs. sham; ^#^*P* < 0.05, ^##^*P* < 0.01 vs. ICH). For panel **D**, *n* = 20 in the sham group, and *n* = 30 in the other groups; for panels **A**, **B**, **E**, and **F**, *n* = 4 in the sham group, and *n* = 6 in the other groups.

To evaluate the protective role of SIRT1 in ICH injury, siRNA was used to knockdown SIRT1 and Western blot and QPCR were used to measure the effectiveness of SIRT1 interference (**Figures 8A–C**). Data show that ICH increased SIRT1, and silencing SIRT1 decreased SIRT1 promoted by ICH. Brain water content and neurobehavioral functions were evaluated 48 h after ICH (**Figures 8D,E**) and brain water content was substantially increased in the ICH group compared with shams in the ipsilateral hemicerebrum. SIRT1 silencing increased brain water content in the ipsilateral hemicerebrum as well compared with the ICH group. Neurological outcome data show that neurological scores in the modified Garcia test were reduced in the ICH group compared with shams (**Figure 8D**). SIRT1 silencing worsened neurobehavioral performance in the adjusted Garcia test compared to the ICH group.

### Histological Assessment 48 h after ICH Injury after SIRT1 Silencing

H&E staining was used to assess morphological alterations in hemorrhagic lesions 48 h after ICH (**Figure 8F**). In contrast to shams, neurocytes in the ICH and ICH + vehicle groups were disordered with shrinking cell bodies, nuclear pyrosis and fragmentation, and there were more red neurons. SIRT1 silencing led to widespread neuronal cell necrosis and decreased neurocytes in the interstitial areas.

### ATP and Mitochondrial DNA Content and Mitochondrial Morphology after ICH Injury after SIRT1 Silencing

**FIGURE 9:**
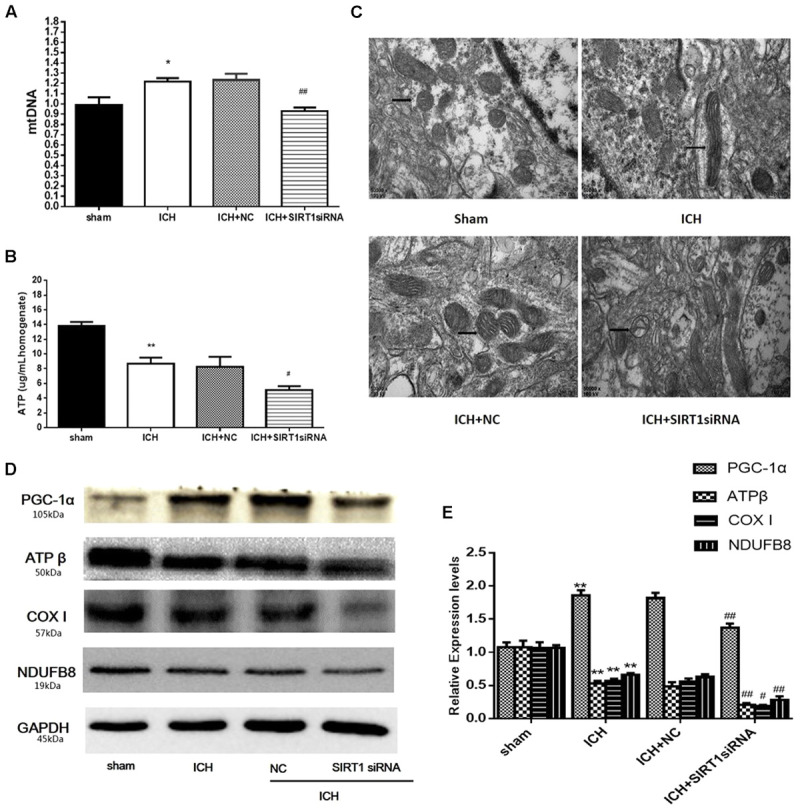
ATP concentration, mitochondrial DNA content, mitochondrial structural and mitochondrial electron transport chain proteins changes after ICH injury following SIRT1 silencing in rats. **(A)** Real-time PCR of the D-loop region of mtDNA in brain tissue at 48 h after ICH. **(B)** Changes in ATP concentration of brain tissue at 48 h after ICH as assessed by high performance liquid chromatography with a variable wavelength detector (HPLC-VWD). **(C)** Mitochondrial structural changes were assessed by projection electron microscopy (50000× magnification). Arrows indicate mitochondria around the hematoma of rats with ICH. **(D)** Representative western blots of ATPβ, COX I, NDUFB8, and PGC-1α. Relative protein band density values were calculated as the ratio of the protein of interest to that of GAPDH. Quantification of **(D)** is shown in **(E)**. Error bars represent mean ± SEM. (^∗^*P* < 0.05, ^∗∗^*P* < 0.01 vs. sham; ^#^*P* < 0.05, ^##^*P* < 0.01 vs. ICH). *n* = 4 in the sham group, and *n* = 6 in the other groups.

ATP was substantially reduced in the ICH group compared with shams, and ATP decreased in ICH + SIRT1 siRNA groups compared with the ICH group (**Figure 9B**). Compared with shams, mtDNA increased in the ICH group and SIRT1 silencing decreased mtDNA compared with the ICH group (**Figure 9A**). Widening of the mitochondrial outer compartment was substantially greater in the ICH group than in shams. After SIRT1 silencing, mitochondrial swelling was enhanced in the mitochondrial outer and inner compartment compared with the ICH group (**Figure 9C**).

### SIRT1 Silencing Decreased PGC-1α and Mitochondrial Electron Transport Chain Proteins after ICH Injury

Data to clarify a protective role of SIRT1 in modulating expression of PGC-1α and mitochondrial electron transport chain proteins suggest that SIRT1 silencing reduced expression of PGC-1α and mitochondrial electron transport chain proteins after ICH (**Figures 9D,E**).

### Expression of Apoptotic Proteins after SIRT1 Silencing after ICH

**FIGURE 10:**
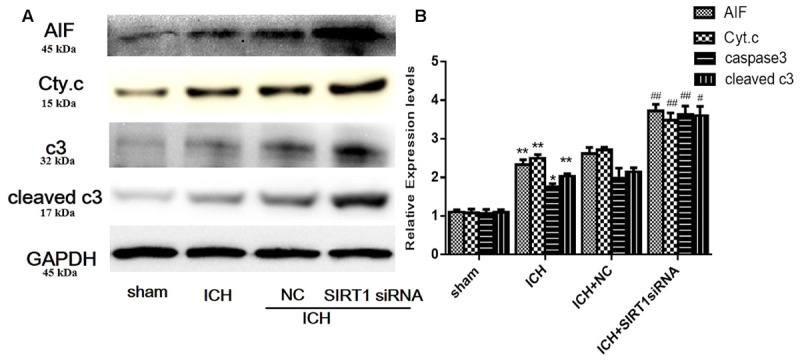
Expression levels of apoptotic proteins after SIRT1 silencing following ICH. **(A)** Representative western blots of AIF, cytochrome c, caspase-3, and cleaved caspase-3. Relative protein band density values were calculated as the ratio of the protein of interest to that of GAPDH. Quantification of **(A)** is shown in **(B)**. Error bars represent mean ± SEM. (^∗^*P* < 0.05, ^∗∗^*P* < 0.01 vs. sham; ^#^*P* < 0.05, ^##^*P* < 0.01 vs. ICH). *n* = 4 in the sham group, and *n* = 6 in the other groups.

We measured apoptotic proteins to evaluate SIRT1 silencing and compared with shams, ICH increased expression of caspase-3 (c3), cleaved caspase-3 (cleaved c3), cytoplasmic release of cytochrome c, and cytoplasmic of AIF (**Figures 10A,B**). SIRT1 silencing increased these effects compared with the ICH group so SIRT1 silencing may exacerbate apoptosis.

## Discussion

Mitochondrial abnormality often causes organ damage and conditions characterized by metabolic inadequacy. Mitochondrial integrity is vital for cell and organ function due to roles in ATP generation, fatty acid and lipid metabolism, signaling pathways, and programmed cell death. Aside from possible treatments for conditions distinguished by mitochondrial dysfunction, few treatments target mitochondria to induce function. Also, there are no effective treatments targeting mitochondrial dysfunction for ICH, a severe stroke subtype that requires therapeutic options.

Here, we report that activation of SIRT1 with SRT1720 significantly reduced brain water content and neurobehavioral deficits and increased recovery of mitochondrial function after ICH. Activation of SIRT1 was linked to increased nuclear SIRT1 and PGC-1α deacetylation, suggesting that the mechanism of action is via a SIRT1/PGC-1α pathway. Moreover, siRNA knockdown of SIRT1 significantly worsened ICH injury in rats. Thus, we propose that activation of the SIRT1/PGC-1α pathway to increase mitochondrial function post-ICH may provide distinctive therapeutic benefits that promote recovery of brain function. Also, we may be able to develop novel therapeutic approaches to treat injury to other organs affected by mitochondrial abnormalities.

After ICH, nuclear SIRT1 expression decreased in brain tissue and increased total SIRT1 expression. Activation of SIRT1 with SRT1720 restored nuclear SIRT1 expression in brain tissue after ICH. Similar results were reported after myocardial infarction (Tong et al., 2013). There are many activators of SIRT1, and most act through AMPK but SRT1720 is a specific activator of SIRT1 and interacts directly with SIRT1 to increase expression and activity. Consistent with a previous study, our results revealed no significant difference in pAMPK between ICH and ICH + SRT1720 groups.

We examined a protective role of activation of SIRT1 in mitochondrial biogenesis in rats after ICH by evaluating differences in functional yield and by assessing specific mitochondrial proteins, mtDNA, and mitochondrial structure. ATPβ, NDUFB8, and COX I are mitochondrial electron transport chain-related proteins important for regulating ATP content. After ICH, although total SIRT1 expression increased, mitochondrial proteins, such as ATPβ, NDUFB8, and COX I, were reduced in the ICH group compared with shams, and this was also reported in acute kidney injury (Funk and Schnellmann, 2012). Recently, numerous studies indicate that SIRT1 function varies with subcellular localization, and subcellular concentration of SIRT1 is critical for determining its activity and substrate availability. The observed decrease in mitochondrial electron transport chain-related proteins after ICH may have resulted from loss of nuclear SIRT1 expression. Consistent with this hypothesis, results showed that activation of SIRT1 could mostly restore mitochondrial proteins with increased expression of nuclear SIRT1 after ICH. Also, cytoplasmic SIRT1 can increase cell susceptibility to death, which is probably due to reduced interactions with nuclear apoptotic mediators such as p53, FOXO, and Ku70 (Jin et al., 2007). Our results showed increased mitochondrial DNA copy number, reduced mitochondrial swelling, and increased expression of cytoplasmic SIRT1 in the ICH + SRT1720 group compared with the ICH group. We also verified that increases in mitochondrial elements correlated with improved mitochondrial yield by assessing ATP biogenesis after activation of SIRT1. These findings are consistent with results of a previous study of isoflavone-promoted mitochondrial biogenesis with SIRT1 initiation (Rasbach and Schnellmann, 2008), and studies indicate that mitochondrial biogenesis was stimulated with resveratrol in other cell types (Lagouge et al., 2006; Csiszar et al., 2009). Thus, these results indicate that activation of SIRT1 exerts an important protective role in mitochondrial dysfunction induced by ICH.

PGC-1α is a primary target for mitochondrial malformation due to its controlling role in modulating metabolic processes and intracellular mitochondrial activity and biogenesis. Increasing PGC-1α or activity can reverse phenotypic consequences of mitochondrial damage. Mitochondrial myopathies can be rescued by transgenic expression of PGC-1α or treatment with peroxisome proliferator-activated receptor agonist bezafibrate, which promotes mitochondrial biogenesis, improves respiratory ability, protects ATP, and lengthens life span (Wenz et al., 2008). Current research shows that promotion of PGC-1α and mitochondrial biogenesis is a pivotal adaptive response to maintain energy and metabolic requirements needed during recovery from particularly severe injury to cells and organs (Rasbach and Schnellmann, 2007; Wang et al., 2008; Yin et al., 2008). As a transcriptional co-activator, deacetylated PGC-1α more efficiently enlists transcription components to promote synthesis of its primary target genes, which have been linked to gluconeogenesis, fatty acid oxidation, and mitochondrial biogenesis (Gerhart-Hines et al., 2007; Rohas et al., 2007; Rodgers et al., 2008). Each of these processes preserves a component of PGC-1α in mitochondrial dysfunction. A previous study showed that SIRT1 is a pivotal activator of PGC-1α in various diseases and that there are two pathways through which SIRT1 regulates PGC-1α: regulating PGC-1α and controlling activity of PGC-1α (Tsuruoka et al., 2012). Tong’s group showed that SIRT1 modulates PGC-1α deacetylation to increase its activity and this can be cardioprotective as PGC-1α functionally interacts with SIRT1 and promotes deacetylation of PGC-1α (Nemoto et al., 2005; Gerhart-Hines et al., 2007; Canto and Auwerx, 2009). Here, we report decreased deacetylated PGC-1α in rats subjected to ICH and activation of SIRT1 prevented this decrease and increased nuclear PGC-1α expression. These results may be explained by increased nuclear localization of SIRT1. In addition, reduced electron transport chain proteins may have resulted from decreased deacetylated PGC-1α. To explore potential mechanisms underlying the protective role of SIRT1 activation, we used siRNA to knockdown PGC-1α. After ICH, PGC-1α knockdown eliminated recovery of mitochondrial electron transport chain protein expression after treatment with SRT1720. Thus, the protective role of SIRT1 activation in mitochondrial biogenesis in rats after ICH is mediated by PGC-1α.

Impairment after ICH is caused by primary and secondary injuries, and neuronal cell death is a major problem. Apoptosis operates through external (death receptor pathway) and internal (mitochondrial pathway) pathways (Elmore, 2007), and pathways converge by initiation of caspase-3. In response to various intracellular apoptotic signals, mitochondria undergo reduction of intrinsic mitochondrial membrane integrity and subsequently release several pro-apoptotic proteins (Otera and Mihara, 2012). Of these proteins, cytosolic cytochrome c complexes with additional pro-apoptotic components to form apoptosomes, which then promote the caspase-dependent proteolytic cascade (Joza et al., 2001). In contrast, AIF participates in caspase-free apoptosis by migrating to the nucleus (Galluzzi et al., 2012). Consistent with these studies, our *in vivo* ICH experiments indicated a significant increase in caspase-3 and mitochondrial-related apoptotic proteins, and SIRT1 activation effectively reduced these proteins. In addition, the protective role of SIRT1 in regulating mitochondria-dependent apoptosis may be achieved by reducing mitochondrial dysfunction. To explore possible mechanisms of SIRT1 in regulating mitochondria-dependent apoptosis, we examined the effects of PGC-1α silencing on mitochondria-dependent apoptotic proteins. Results showed that expression of cytochrome c and AIF was higher in the ICH + SRT1720 + PGC-1α siRNA group than in the ICH + SRT1720 group. Thus, a critical protective role of SIRT1 in mitochondria-dependent apoptosis is mediated by PGC-1α.

To confirm the role of SIRT1 in ICH injuries, siRNA was used to knockdown SIRT1 and this decreased expression of PGC-1α and increased brain water content, neurobehavioral deficits, mitochondrial dysfunction, and mitochondria-dependent apoptosis. These data verify a protective role of SIRT1 in ICH-induced mitochondrial dysfunction and apoptosis. Also, the protective effects of SIRT1 may be mediated by the SIRT1/PGC-1α pathway.

## Conclusion

SIRT1 contributes to ICH by protecting against mitochondrial abnormalities and mitochondrial-dependent cell death by enhancing PGC-1α activity via modulation of deacetylation and expression of PGC-1α. Collectively, our data suggest that activation of the SIRT1/PGC-1α pathway may be a target for new therapies to prevent mitochondrial dysfunction and apoptosis in patients with ICH.

## Author Contributions

YaZ and YoZ conceptualized the experiments. SW, SY, and YL performed the experiments. YaZ and YoZ analyzed the data. YaZ wrote the paper, and SY and YoZ edited the manuscript. All authors reviewed the manuscript.

## Conflict of Interest Statement

The authors declare that the research was conducted in the absence of any commercial or financial relationships that could be construed as a potential conflict of interest.
