# Correction: SIRT1/PGC-1α signaling promotes mitochondrial functional recovery and reduces apoptosis after intracerebral hemorrhage in rats

**DOI:** 10.3389/fnmol.2026.1903556

**Published:** 2026-07-16

**Authors:** Yang Zhou, Shaohua Wang, Yixin Li, Shanshan Yu, Yong Zhao

**Affiliations:** 1 Department of Pathology, Chongqing Medical University, Chongqing, China; 2 Institute of Neuroscience, Chongqing Medical University, Chongqing, China

**Keywords:** SIRT1, PGC-1α, intracerebral hemorrhage, mitochondrial biogenesis, apoptosis

In the published article, there was an error in Figure 4. In Figure 4C, during the figure assembly process, the same image was erroneously added in the collage and should have not been included.

The corrected Figure 4 appears below.

**Figure 4:**
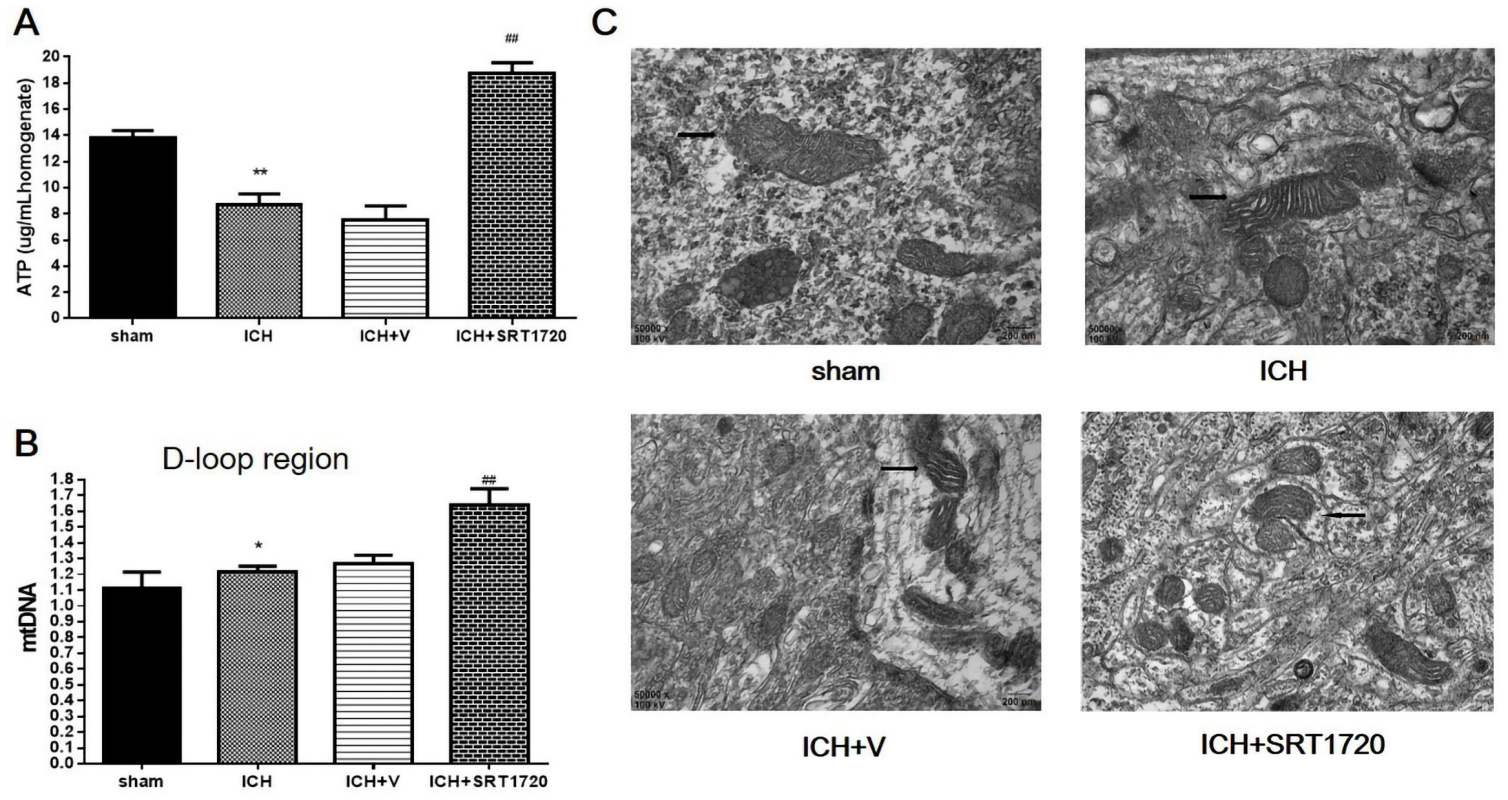
ATP concentration, mitochondrial DNA content, and mitochondrial structural changes after ICH injury after SIRT1 activation in rats. **(A)** Changes in ATP concentration in the brain tissue 48 h after ICH assessed with HPLC with a variable wavelength detector (HPLC-VWD). **(B)** Real-time PCR of the D-loop area of mtDNA in brain tissue 48 h after ICH. **(C)** Mitochondrial structural changes assessed by projection electron microscopy (50,000x). Arrows indicate mitochondria around hematomas of ICH rats. Error bars represent means ± SEM. (^*^P < 0.05, ^**^P < 0.01 vs. sham; ^##^*P* < 0.01 vs. ICH). *n* = 4 in sham group, and n = 6 in other groups.

The original version of this article has been updated.

